# Memristive Artificial Synapses for Neuromorphic Computing

**DOI:** 10.1007/s40820-021-00618-2

**Published:** 2021-03-06

**Authors:** Wen Huang, Xuwen Xia, Chen Zhu, Parker Steichen, Weidong Quan, Weiwei Mao, Jianping Yang, Liang Chu, Xing’ao Li

**Affiliations:** 1 New Energy Technology Engineering Laboratory of Jiangsu Province and School of Science, Nanjing University of Posts and Telecommunications (NJUPT), Nanjing, 210023 People’s Republic of China; 2 Key Laboratory for Organic Electronics and Information Displays and Institute of Advanced Materials, Jiangsu National Synergistic Innovation Center for Advanced Materials, School of Materials Science and Engineering, Nanjing University of Posts and Telecommunications (NUPT), 9 Wenyuan Road, Nanjing, 210023 People’s Republic of China; 3 College of Electronic and Optical Engineering and College of Microelectronics, Nanjing University of Posts and Telecommunications (NJUPT), Nanjing, 210023 People’s Republic of China; 4 Department of Materials Science and Engineering, University of Washington, Seattle, WA 98195-2120 USA

**Keywords:** Synaptic devices, Neuromorphic computing, Electrical pulses, Optical pulses, Photoelectric synergetic effects

## Abstract

Neuromorphic computing simulates the operation of biological brain function for information processing and can potentially solve the bottleneck of the von Neumann architecture. This computing is realized based on memristive hardware neural networks in which synaptic devices that mimic biological synapses of the brain are the primary units. Mimicking synaptic functions with these devices is critical in neuromorphic systems. In the last decade, electrical and optical signals have been incorporated into the synaptic devices and promoted the simulation of various synaptic functions. In this review, these devices are discussed by categorizing them into electrically stimulated, optically stimulated, and photoelectric synergetic synaptic devices based on stimulation of electrical and optical signals. The working mechanisms of the devices are analyzed in detail. This is followed by a discussion of the progress in mimicking synaptic functions. In addition, existing application scenarios of various synaptic devices are outlined. Furthermore, the performances and future development of the synaptic devices that could be significant for building efficient neuromorphic systems are prospected.

## Highlights

- Synaptic devices that mimic synaptic functions are discussed by categorizing them into electrically stimulated, optically stimulated, and photoelectric synergetic synaptic devices based on stimulation of electrical and optical signals.
- The working mechanisms, progress, and application scenarios of synaptic devices based on electrical and optical signals are compared and analyzed.
- The performances and future development of various synaptic devices that could be significant for building efficient neuromorphic systems are prospected.

## Graphical abstract

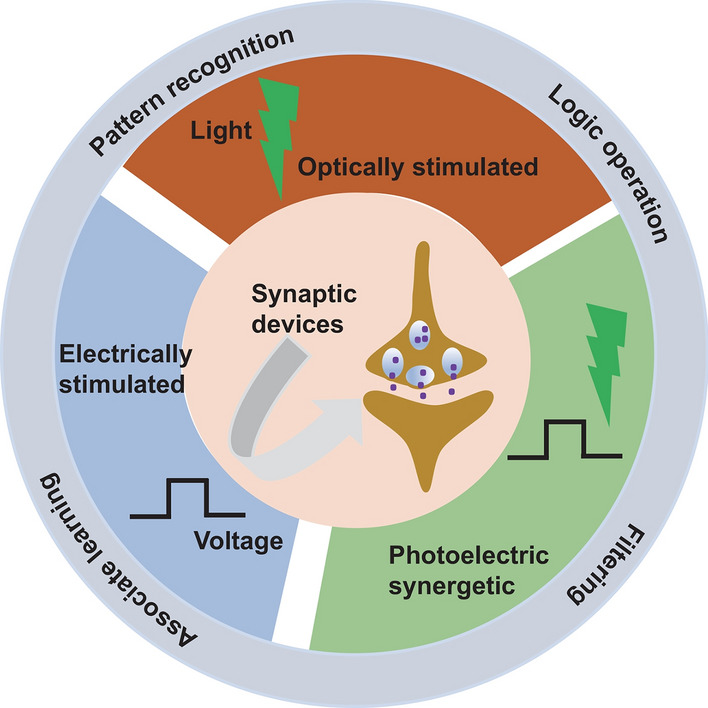

## Introduction

**Fig. 1:**
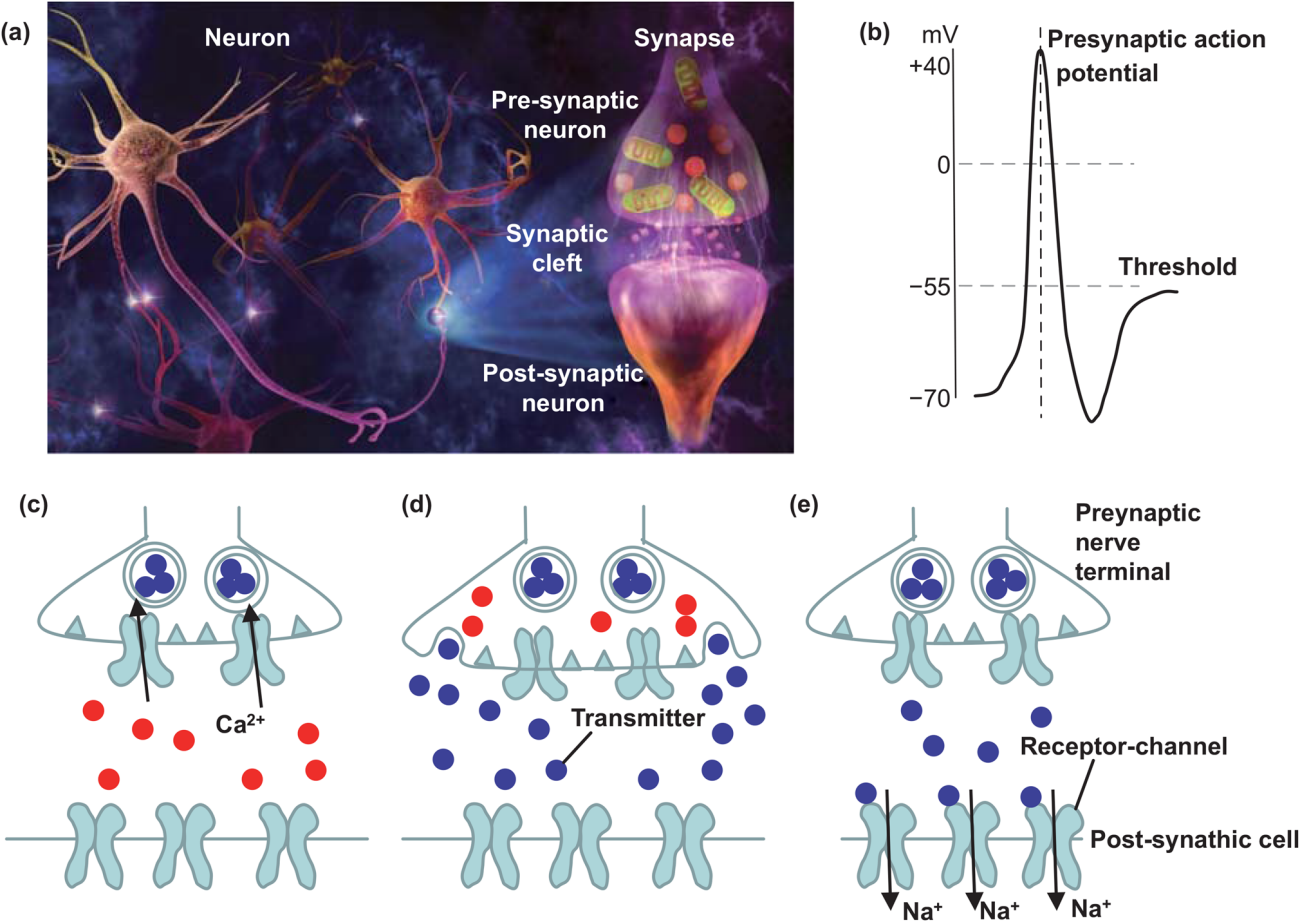
**a** Schematic of biological neurons and synapse. Reproduced with permission from Ref. [4]. **b** Illustration of an action potential arriving at the presynaptic terminal. **c** Opening of voltage-gated Ca^2+^ channels at the active zone in response to the action potential. **d** Release of neurotransmitters in the vesicles into the synaptic cleft. **e** Diffusion of neurotransmitters across the cleft and binding with specific receptors on the postsynaptic terminal. **b**–**e** Adapted with permission from Ref. [10]

In the past half century, computers based on the traditional von Neumann architecture have achieved great progress given their powerful capabilities to deal with computational problems [1]. However, the processors and the memory are physically separated, causing a series of problems such as slow calculation speed and high energy consumption. In addition, the von Neumann computing mechanism runs in accordance with a specific program, rendering the self-evolution and timely resolution of problems impossible [2]. Furthermore, Moore’s law-based device scaling for improving computing abilities has been significantly slowing down in recent years [3]. These shortcomings limit the further development of silicon CMOS-based computing hardware. With the rise of big data, the Internet of Things, and artificial intelligence, the demand for low-energy and highly adaptable computing has gradually increased. The human brain has a neural network circuit composed of 10^11^ neurons and 10^15^ synapses (Fig. 1a) [4, 5]. With the distributed and parallel operations in its loop, the human brain has strong memory space and high-speed computing abilities [6]. It can maintain a low power consumption during operation, thus achieving the advantage of adapting to the outside world. Inspired by the human brain, an artificial neural network (ANN) is being built to successfully realize brain-like computing and is considered the most effective solution to the von Neumann bottleneck [7].

Brain-like computing for learning and information processing utilizes the plasticity of neural synapses in the human brain neural network [8]. Each synapse is composed of the presynaptic terminal, postsynaptic terminal, and the narrow gap between the two terminals, that is, the synaptic cleft (Fig. 1a) [9]. The synapse is where neurons are functionally connected and is the key component for information transmission. As shown in Fig. 1b–e, when a presynaptic action potential reaches the front of a synapse, the calcium (Ca^2+^) channel controlled by the potential opens [10]. Ca^2+^ enters the presynaptic membrane and causes the opening of vesicles containing neurotransmitters. Neurotransmitters are then released into the synaptic cleft and bind to the receptors of the postsynaptic membrane, causing the opening or closing of Na^+^ channels of the posterior membrane. As a result, the conductivity of the synapses changes, which correspond to the behaviors of the excitatory/inhibitory postsynaptic current (EPSC/IPSC). This process is called synaptic plasticity and plays a critical role in the information transmission in the human neural network.

Synaptic plasticity is the experience-dependent change in the connection strength between neurons and is well described by the Hebbian theory [5, 6, 11]. This plasticity comes in different types depending on the shape of external pulses [12]. The different types include short-term potentiation/depression (STP/STD) such as paired pulse facilitation (PPF)/paired pulse depression (PPD) and spike-number-dependent plasticity (SNDP), long-term potentiation/depression (LTP/LTD), spike-rate-dependent plasticity (SRDP), and spike-timing-dependent plasticity (STDP). STP/STD is generally believed to be related to the recognition and processing of external signals by the human brain. This type of synaptic plasticity mainly relies on biochemical changes in neurons, wherein the recognized information is easily forgotten. LTP/LTD is related to human brain learning and memory. This type of synaptic plasticity usually includes changes in structure and gene expression; the memory to external information can last for several months or longer. The transition from short-term to long-term potentiation and depression can occur when the synapse is stimulated by continuous external pulses, which ensures the realization of memory in the human brain. The above-mentioned behaviors can also be realized by changing the amplitude of the spikes, which is called spike-amplitude-dependent plasticity (SADP). SRDP reflects the synaptic plasticity generated by external pulses at different frequencies. This type of synaptic plasticity is one of the most important synaptic functions in the cognitive behavior of the brain. STDP depends on the sequence and interval of external signal pulses arriving at the presynaptic and postsynaptic terminals of the synapse. When the time sequence changes for the signals arriving at both terminals, the synapse weight can change from potentiation (depression) to depression (potentiation). This phenomenon is called asymmetric STDP, otherwise it is called symmetric STDP in the brain. STDP is considered to be the most important learning mechanism of Hebbian theory and plays an important role in information coding, learning, and memory.

Besides, given that information is transmitted between neurons in the brain neural network through synapses [6], the construction of synaptic devices mimicking above synaptic functions is crucial to the development of ANNs. Synaptic behaviors of these devices are similar to brain synapses. When a synaptic device receives an external stimulus, the synaptic weight will change, but it will not immediately return to the initial state. However, when the synapse device is continuously stimulated, its weight will continually change, showing a cumulative effect that can be memorized. Thus, synaptic devices have been constructed based on these properties of various functional material-based memristive systems and studied the synaptic behaviors in response to external stimulation signals [13]. These signals mainly include electrical and optical pulses, which exhibit many advantages in regulating physical properties of these material-based devices and thereby mimicking synaptic functions [2, 4, 12, 14]. In the work, we discuss these synaptic devices by categorizing them into electrically stimulated, optically stimulated, and photoelectric synergetic synaptic devices. Working mechanisms, progress, applications and several perspectives are presented.

## Classification of Synaptic Devices

Different synaptic devices can be classified in accordance with different standards. For example, based on the working mechanism, synaptic devices can be divided into ion migration (electrical pulses) [15], ferroelectric (electrical pulses) [16], phase change (including electrical pulses and optical pulses) [17, 18], charge capture and release (including electrical pulses and optical pulses) types [19, 20], and redox mechanisms [21, 22]. These materials include metal oxide, semiconductors, phase-change materials and perovskite, etc. Based on their structure type, synaptic devices can be divided into two-terminal and transistor-type synaptic devices [23, 24]. The electrode port can be considered a neuron connected to a synapse or a port for signal stimulation. Two-terminal synaptic devices can be easily integrated, whereas transistor-type synaptic devices are difficult to integrate but can well regulate the electrical behaviors through the gate voltages. Based on the stimulation types of synaptic devices, neurosynaptic devices are mainly divided into electrically stimulated and optically stimulated synaptic devices [25, 26]. In addition, photoelectric synergetic synaptic devices that coordinate optical and electrical pulses have emerged as well [27].

### Electrically Stimulated Synaptic Devices

Electrically stimulated synaptic devices mainly use electrical pulses as stimulus signals to change their conductivity, that is, synaptic weights, and realize mimicking of various synaptic functions by changing the stimulation pulse width, voltage amplitude, frequency, number, and so on [28, 29]. One study also reported the utilization of electrical pulses that regulate the luminescence performance of devices to mimic synaptic functions and has generated great interest [30]. Ideal electrically stimulated synaptic devices are expected to possess memory on synaptic weight changes. Thus, the design and preparation of synaptic devices must consider a device structure which can memorize resistance states achieved. Memristor is a kind of resistive random-access memory (RRAM) [31, 32]. When stimulated by external electrical pulses, its resistance state changes based on different mechanisms [33]. When the electric field is withdrawn, the previous resistance value can be maintained, and the memory effect can be realized. This behavior is similar to the memory function of biological synapses [34]. Since the advent of memristors, researchers have used them as synaptic devices to mimic biological synaptic functions [2, 35, 36]. The current research on the application of electric memristive systems in the field of synaptic devices mainly includes ion migration [24, 33, 37], ferroelectric [16, 38–40], phase-change [41, 42, 74], and carrier-capture and release types [19]. This section mainly focuses on these device types.

#### Ion-Migration-Type Synaptic Devices

Ion-migration-type memristors mainly use external electrical pulses to trigger the movement of metal ions or oxygen (halogen) vacancies, leading to the formation of conductive filaments [33]. With the application of reverse electrical pulses, metal ions or oxygen (halogen) vacancies move in the opposite direction, and the filament breaks. Researchers use this process to control changes in the electrical conductivity, thereby mimicking biological synaptic functions [14].

**Fig. 2:**
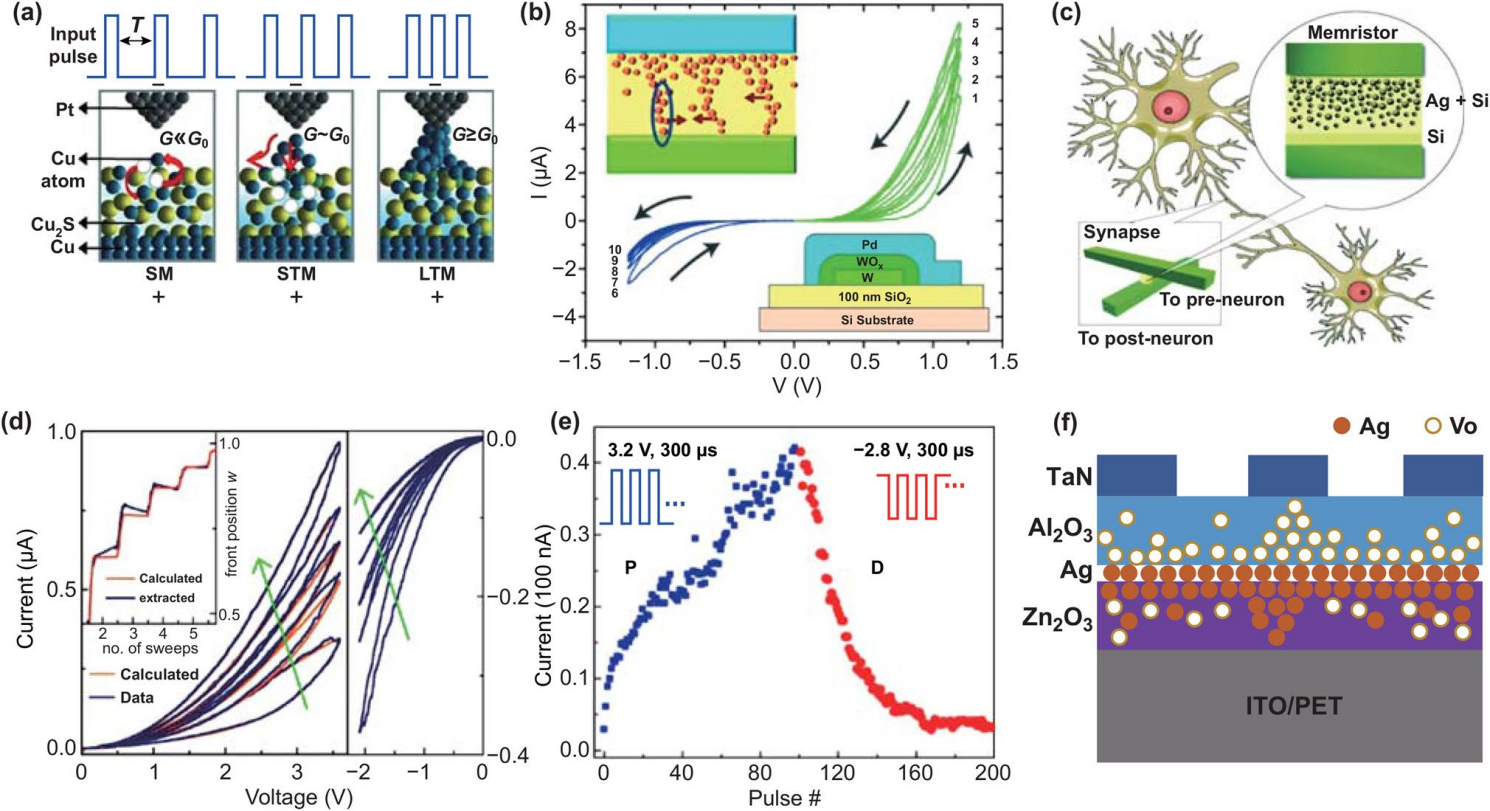
**a** Atomic-switch-based synaptic devices. Reproduced with permission from Ref. [43]. **b** Changes in conductance versus the increase in the sweep number of oxygen vacancy (or oxygen ion) diffusion-based synaptic device. Reproduced with permission from Ref. [47]. **c** Schematic of Ag-migration-memristor-based synapse device. **d** Measured (blue lines) and calculated (orange lines) *I–V* characteristics of the memristor. Inset: calculated and extracted values of Ag front position w versus the number of sweeps. **e** Current values of the devices in response to external potentiation and depression pulses. **c**–**e** Reproduced with permission from Ref. [61]. **f** Schematics of the device structure and the corresponding mechanism. Adapted from Ref. [60]

Metal ion migration devices are some of the most common memristors for mimicking synaptic functions. One category is the atomic switch model based on a Cu_2_S system (Fig. 2a), as can be seen in the work of Nayak et al. [43]. The device worked in three states in response to external pulses, corresponding to sensor memory (SM), short-term memory (STM), and long-term memory (LTM). In the SM state, a small precipitation of copper (Cu) atoms occurred in the gap in response to external pulses. The resulting increase in conductivity was quite small. With the increase in pulse amplitude and frequency, more Cu atoms were driven to the gap, gradually increasing the conductivity. Finally, STM and LTM states were formed, respectively. In the same manner, Ohno et al. [37] constructed synaptic devices using Ag_2_S. These systems have a common feature of artificially constructing a nanogap between the active layer and Pt electrode under the control of a scanning tunneling microscope and synaptic functions are mimicked during the operations. The other category is based on the structure of an active electrode silver such as Ag/dielectric/an inactive electrode [33, 36, 44, 45]. An applied electrical pulse was used to regulate the Ag movement and finally change the electrical performance of the device [45]. Synaptic functions were mimicked successfully during the formation and rupture of the filaments in response to external electrical pulses.

Oxide-based memristors regulate the movement of oxygen vacancies through electrical pulses [33], thereby controlling the formation and breaking of conductive filaments to mimic synaptic functions [46]. In recent years, these oxide-based memristors have received extensive attention. Chang et al. [47] studied the memristor of the WO_x_ system and successfully implemented the mimicking of the synaptic functions (Fig. 2b). In the case of external bias pulses, oxygen vacancies in the device were redistributed, mimicking synaptic functions through the formation and rupture of the filaments. This process is similar to migration of halide ions in perovskites for mimicking various synaptic functions [48]. Resistive switching during the ion migration can also be attributed to the redox processes occurring at the interface between the active materials and an oxidizable electrode as well as in the channels [21, 49], which could mimic the synaptic functions as well. Besides, organic materials, which have properties of low cost, exibility, good environmental stability, and simple fabrication procedures, have been intensively explored for neuromorphic applications. The synaptic weights are mainly controlled by the ion migrations in these material-based structures [50–57]. For example, Lee et al. [53] designed a ONW (organic nanowire) synaptic transistor. Ions penetrated the PEO sheath or even the P3HT core under the spikes, and conductivity was changed accordingly. Plasticity was then induced during this process. Zhu et al. [50] built a transistor-structure-based synaptic device, in which ITO was used as the channel and organic proton-conducting electrolytes as the gate. Under the positive electrical pulses at the gate, protons (charged ions) accumulated at the interface of the electrolytes and channel, which can control the distribution of electrons in the channel and mimic LTP synaptic function. The LTD synaptic function was also realized under the negative electrical pulses.

The above-discussed synaptic devices are mainly based on the migration of various ions. The size can be decreased to the nanometer limit region, which is beneficial to their integration [32]. When connected, the electrical properties of synaptic devices may increase significantly, and their dynamic range is large [44]. However, the relationship between conductivity and stimulation times is nonlinear, which does not meet the requirements of excellent linearity for synaptic weight change versus the stimulation for neural network online learning [58, 59]. Therefore, researchers have sputtered metal particles in the active layers or added ion blocking layers during preparation [60], as discussed below, which can retain the change in synaptic weight with relatively good linearity and avoid sudden changes in electrical properties. For example, Jo et al. [61] used the co-sputtering of Ag and Si method to incorporate a certain proportion of Ag particles into the silicon matrix (Fig. 2c). From the *I*–*V* curves in Fig. 2d [61], the conductivity exhibited hysteresis for each sweep under both forward bias and reverse bias, indicating that voltage can cause the back-and-forth movement of Ag nanoparticles (NPs) in the synaptic device. Given this condition (Fig. 2e), LTP and LTD synaptic functions were successfully mimicked in response to continuous external pulses. Meanwhile, the asymmetric STDP synaptic function was also realized by changing the time interval Δ*t* and sequence of the applied pulses arriving at the presynaptic and postsynaptic terminals. Similarly, Wang et al. [60] used atomic-beam deposition and spin-coating techniques to prepare a vertical two-terminal synaptic device based on a TaN/Al_2_O_3_:Ag:ZnO/ITO RRAM device on a flexible substrate (Fig. 2f). The mechanism of the resistance switch in this device includes migrations of oxygen ions and Ag ions. Through the introduction of electrical pulses, the movement of Ag ions to ZnO was regulated to form a conductive wire. When a reverse electrical pulse was applied, the Ag gradually returned to the interface between Al_2_O_3_ and ZnO, breaking the conductive wire. The embedded Ag nanoparticles provide a path for the filament formation, reducing the variability in resistances from more than 160% to 30% between high and low resistance states. This provides the possibility for the improvement in the LTP-update linearity. Similarly, in an oxide-based synaptic device TiN/TaO_x_/Pt, an SiO_2_ oxygen vacancy (ion) barrier layer was added between TiN and TaO_x_ [46]. This suppressed the migration of oxygen ions and significantly improved the linearity of the LTP property.

#### Ferroelectric Synaptic Devices

Under the influence of an applied electric field, the positive and negative charges inside ferroelectric materials separate to form a polarized charge [62–64]. When the applied electric field is removed, the polarized state will not disappear immediately. This function triggers the appearance of hysteresis loops of the electric polarization versus electric field, exhibiting a memristive property. This is a basic characteristic of ferroelectric materials. The ferroelectric region in ferroelectric materials is composed of several iron domains. Under the stimulation of electrical pulses, the device is polarized, and its polarization is determined by the average polarization degree of each iron domain. In the case of continuous electrical stimulation, the polarization of ferroelectric materials leads to gradual changes in electrical properties of the devices [65]. Such properties have been utilized in the study of synaptic devices [38, 66].

**Fig. 3:**
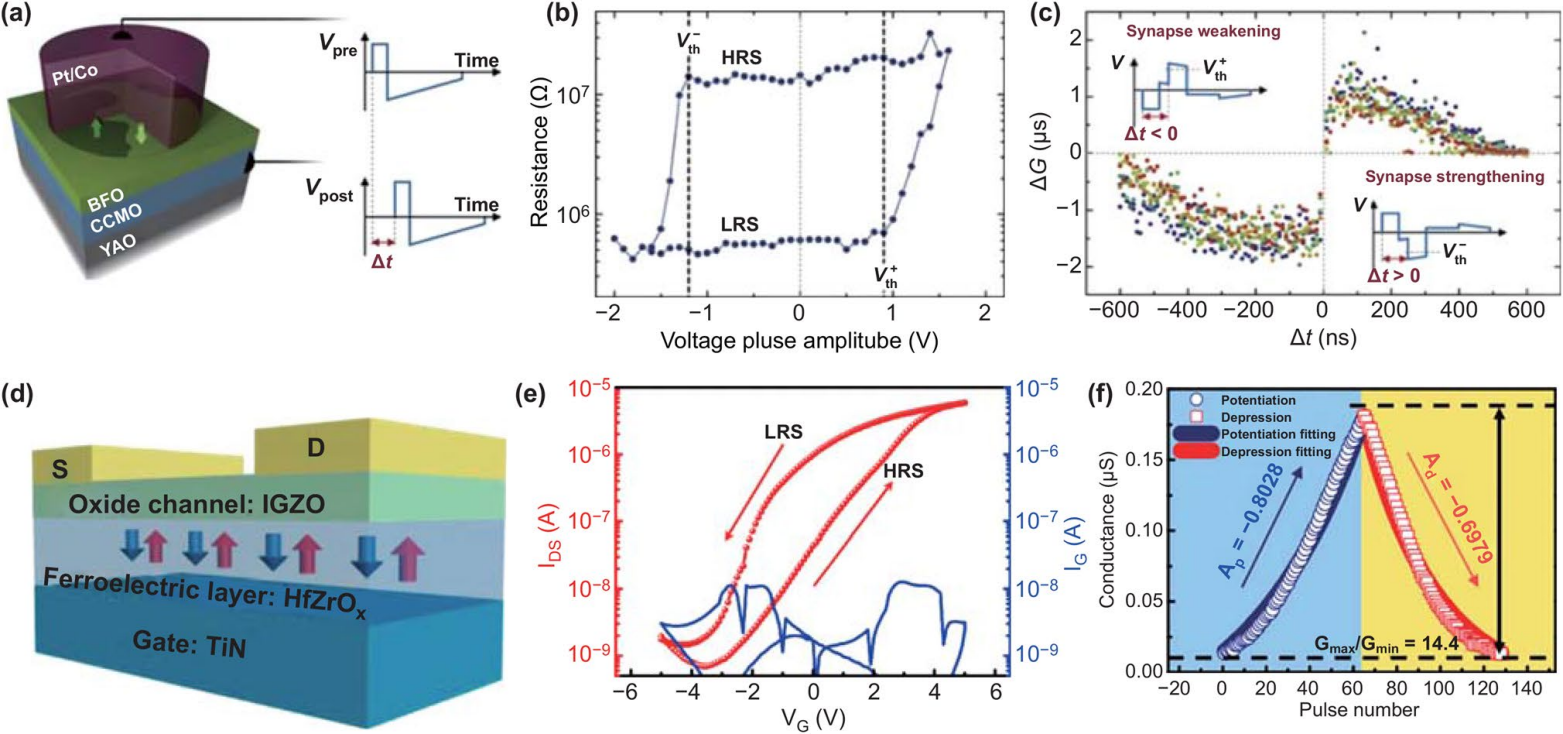
**a** Schematic of a ferroelectric memristor in which a ferroelectric tunnel barrier of BiFeO_3_ is sandwiched between (Ca, Ce) MnO_3_ and Pt/Co. **b** Single-pulse hysteresis loop of resistance versus voltage pulse amplitude. **c** STDP synaptic function mimicked in the device. **a**–**c** Reproduced with permission from Ref. [39]. **d** Schematic of a ferroelectric thin-film (HfZrO_x_) transistor-based synaptic device. **e** *I-V* curves of the conductance of channel versus *V*_G_. **f** Conductance changes of the device in response to successive pulses. **d**–**f** Reproduced with permission from Ref. [16]

Soren et al. [39] constructed a two-terminal synaptic device based on a ferroelectric tunnel junction (FTJ), the layout of which is CaCeMnO_3_ (CCMO)/BiFeO_3_(BFO)/Co (Fig. 3a). BFO is a ferroelectric thin film in the device, while CCMO and Co are used as the bottom and top electrodes, respectively. Figure 3b illustrates the observed hysteresis of resistance versus electrical bias. There is resistance change between low resistance (LRS) and high resistance (HRS) states. Based on this property, the STDP synaptic function was successfully realized by designing the corresponding waveforms of the electrical spikes arriving at presynapses and postsynapses (Fig. 3c). Recently, a two-terminal Pt/BaTiO_3_/Nb-doped SrTiO_3_ FTJ was built by Ge et al. [66] for mimicking synaptic functions. The current of the domain exhibited an increase upon application of positive electric bias pules, which indicated a lower resistance for the downward-polarized domain in the devices. The tendency of the conductance change was opposite to the work of Soren et al. STDP synaptic function was successfully mimicked with femtojoule (fJ) energy consumption, which is comparable with that of human brains (1–10 fJ). Due to the scalability of ferroelectric films, the above two-terminal devices are simple and easy to integrate. These characteristics promote the application of two-terminal ferroelectric synaptic devices in neuromorphic computing. However, the linearity of the LTP properties was poor and needs further investigations to be improved.

In a transistor structure based on the hybrid of a ferroelectric material and other oxide semiconductor materials, the polarization charge generated in the ferroelectric material by applying a gate voltage can regulate the carriers’ concentration in the channel of an oxide semiconductor materials nearby. The electrical performance of the device is thereby successfully regulated to mimic biological synaptic functions [16, 67]. Figure 3d illustrates the ferroelectric transistor-type synaptic device constructed by Kim et al. [16]. From the observed hysteresis effect in the *I–V* curve (Fig. 3e), it indicated charge accumulation and disappearance occurred at the interface between channel IGZO and the ferroelectric material HfZrO_x_, respectively, due to the polarization of HfZrO_x_ under the applied electric field (*V*_G_). LTP and LTD synaptic behaviors were mimicked based on this property. The synaptic weight of the synaptic device changes linearly with the increase in the number of pulse stimulations. This condition is highly conducive to effective online learning efficiency of artificial neural networks [58]. This principle is exactly the same as the work of Li et al. [67], in which PbZr_0.2_Ti_0.8_O_3_ (PZT) was used as the ferroelectric layer in the device. A linear-update LTP was also observed in the device, with a digit recognition accuracy of 94.4% in the neural network. However, a transistor-based device is difficult to shrink and is not conducive to device integration. A large pulse voltage is needed to change the conductivity, and the energy consumption is high for most transistor-based ferroelectric synaptic devices [16].

#### Phase-Change Synaptic Devices

Resistive switching can be based on the phase-change materials between two states: amorphous and crystalline. When a sufficiently large electrical pulse is applied to generate the heat required, the material undergoes a phase transition from an amorphous state (high resistance) to polycrystalline or monocrystalline state (low resistance) [68–70]. Using the difference in the resistance between these states, a series of synaptic functions are mimicked in the corresponding structure [41, 42, 71–74].

**Fig. 4:**
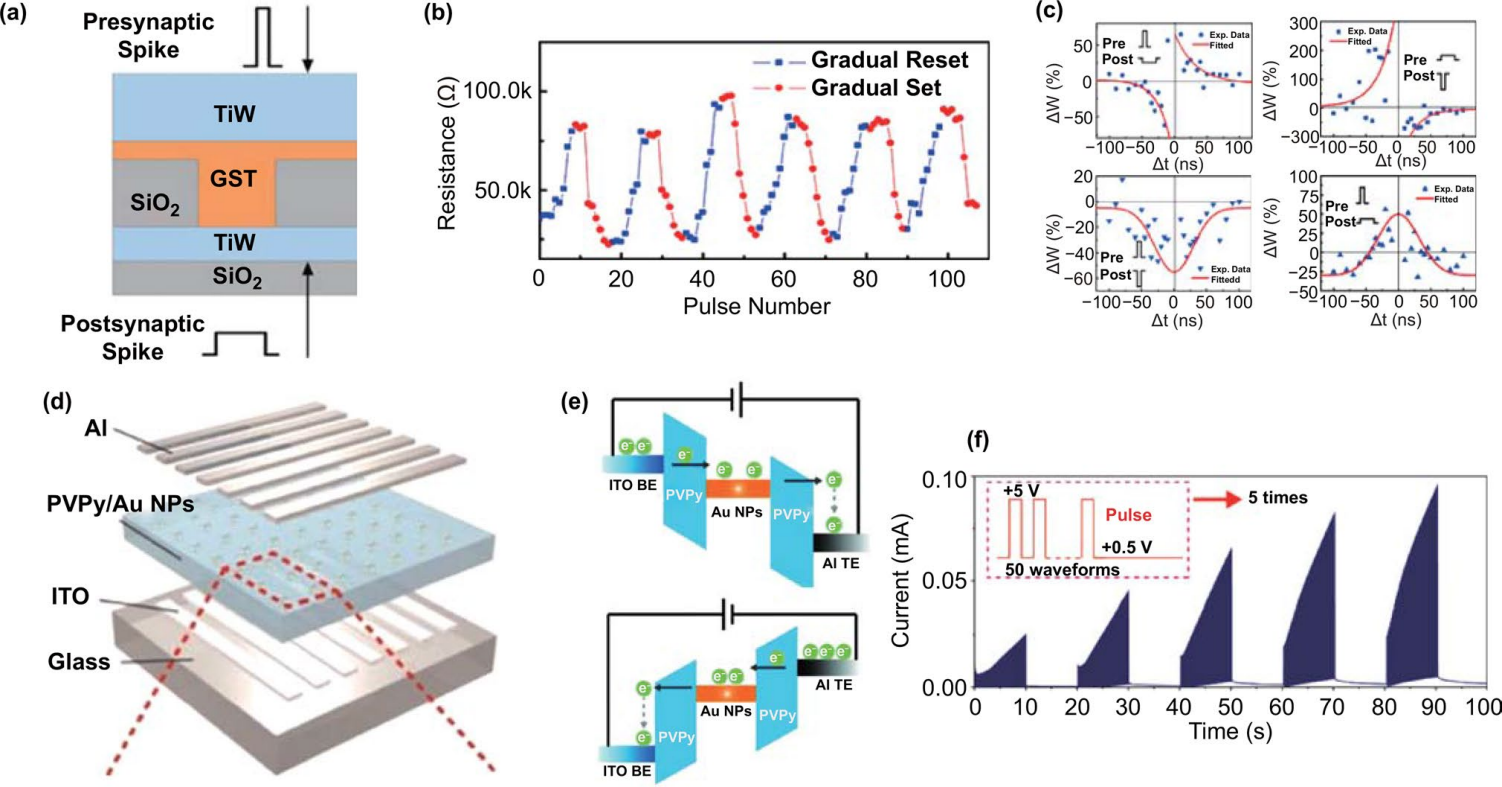
**a** Schematic of the electronic synaptic device based on phase-change memory (PCM) materials of Ge_2_Sb_2_Te_5_ (GST). **b** Resistance changes in response to the external electrical pulses for the gradual reset and set. **c** STDP synaptic functions realized in the device. **a**–**c** Reproduced with permission from Ref. [42]. **d** Crossbar structure of ITO/poly(4-vinylpyridine) (PVPy)–Au NP/Al RRAM device. **e** SET (top) and RESET processing (bottom) of the device. **f** Time-dependent synaptic changes of 50 consecutive pulses. **d**–**f** Reproduced with permission from Ref. [78]

Zhong et al. [42] reported a synaptic device based on Ge_2_Sb_2_Te_5_ (GST) phase-change material (Fig. 4a). When the device was reset by using the electrical pulses with increasing amplitude in the range of 1.5 to 2 V with 50 mV voltage steps, it underwent amorphization, and the conductivity gradually became poor (Fig. 4b). In the setting process, the resistance decreased using pulses with the increasing amplitude from 0.75 to 1.25 V with 50 mV steps, and the conductivity improved consistently. The change in the multi-resistance states of this device is related to the mixture of crystalline and amorphous GST materials. An external bias causes an unbalanced temperature distribution, resulting in a change in the volume ratio of crystalline to amorphous phase in the device and a different resistance state. Based on this, four kinds of STDP learning rules have been successfully implemented in these synaptic devices by changing the time interval and sequence of the pules reaching the presynaptic and postsynaptic terminals of the device (Fig. 4c). The realization of STDP function in the device lays a solid foundation for image or digital recognition. However, the inherent unipolar switching property, due to their thermal-effect switching mechanism, leads to a high reset current, and thus, the power consumption is usually high. Furthermore, the number of middle states for the long-term plasticity and its linearity is very limited, which influence the recognition resolution in the neuromorphic computing.

Considering the limited number of the middle states during operation, Ren et al. [72] constructed a T-shaped synaptic device based on oxygen(O)–titanium(Ti)–antimony(Sb)–tellurium(Te) (OTST) materials. The resistance state of the device changed with the pulse voltage at different pulse widths. In this structure, the bond formed by O and Ti caused a slow crystallization rate, which ensured the possibility of improved control of the setting process. The number of intermediate resistance states of the OTST cell then increased to 220, which provided a resolution close to 8 bits, 7 times higher than the minimum qualified resolution of 5 bits. Meanwhile, a good linear relationship with the increase in electrical pulse number during the setting process was achieved. However, the device exhibited a weak nonlinearity property during the RESET process, resulting from the non-cumulative nature of the melt quench. The nonlinearity property of the plasticity changes will lead to a decrease in the computing accuracy and is not expected in the neural network. To overcome the challenge of the nonlinearity property in neuromorphic computing, the 2-PCM synapse has been proposed by Suri et al. [74], using the cumulative SET operation to fulfill the long-term potentiation and long-term depression in the neural network. The linear LTP in the SET process of the OTST cell enhanced the device’s accuracy of cognitive learning to external affairs in the neural network and demonstrated its potential as a candidate synaptic device.

#### Capture and Release of Carriers

Electrical pulses can induce the movement of charged particles (proton, electrons and holes) in the materials. These carriers can then be captured by various trap centers [75]. Mimicking of synaptic functions can be realized based on the process of carriers capture and release [76, 77]. The energy consumption based on this mechanism is expected to be lower than that based on ions migration and phase transition, in which high power supplies are usually needed. Zhang et al. [78] fabricated a two-terminal device based on the hybrid of PVPy(polyvinylpyrrolidone)–Au NPs (gold nanoparticles) (Fig. 4d). Au NPs play the role of electron capture centers. Under electrical stimulation, the capture and release of electrons by Au NPs in the PVPy–Au hybrid film realized the regulation of electrical conductivity in the device (Fig. 4e). Different synaptic functions, including PPF and SRDP, were then mimicked successfully. It is interesting to observe that the current of stabilized state after consecutive pulses is higher than that of last one (Fig. 4f), indicating that the synaptic weight is increasing versus the accumulation of learning times. These phenomena are consistent with the learning process of a human’s brain, in which the relearning process is becoming more and more quick. Besides, the advantage of two-terminal devices is that it is easy to build a crossbar-array structure, which exhibits the potential to be an important component in constructing next-generation neuromorphic systems. However, the inhibitory synaptic current was not achieved in the device.

In view of this, Au NPs–organic memory field-effect transistor (NOMFET) was constructed for mimicking synaptic functions [19, 79]. In this structure, the Au NPs were mainly embedded into the interface between an organic semiconductor (pentacene) and SiO_2_/Si and were used as nanoscale capacitors to store the holes. When electrical spikes were applied from the back gate, holes in the pentacene were trapped and released by the Au NPs. The conductance of pentacene was then regulated, and various synaptic functions including LTD and asymmetry STDP were successfully realized. Similarly, Sarkar et al. [76] constructed a transistor-type synaptic device based on indium phosphide (InP) nanowires. The carriers captured and released by interfacial traps in the MOS structure successfully modulated the channel conductance and mimicked synaptic functions including IPSC and LTD. These advances improve the potential for large applications of filed effect transistors as synaptic devices based on charges capture and release in neuromorphic computing.

We would like to mention that 2D material-based devices have memristive properties as well [80–83]. They are successfully used as synaptic devices based on the above-mentioned mechanisms [84]. For example, due to the movement of metal ions under the electrical pulses, a conductive bridge was formed through filling the boron vacancies in 2D hexagonal-boron nitride (h-BN) materials for mimicking synaptic functions [85, 86]; the migration of Li^+^ ions caused by electrical pulses in 2D MoS_2_ leaded to reversible modulation of the 2H (semiconductor) -1 T′ (metal) phase, and the conductivity was regulated during this process [87]; conductance controllability of the van der Waals (vdW)–hybrid synapse based on WSe_2_ (for hole transport) and MoS_2_ (for electron transport) was achieved by utilizing only electron-trapping phenomenon in the weight control layer formed on h-BN [88]; the native phosphorus oxide that can trap electrons formed together with anisotropic black phosphorus provides a natural oxide/semiconductor heterostructure for mimicking synaptic functions [89]. Carrier dynamics and conductivity can be controlled either at internal layers or at the interface in these 2D materials. Due to their high carrier mobility, these materials have attracted people’s attention in mimicking synaptic functions in recent years. This opens up a whole new path for low-energy consumption and high-speed synaptic electronics and hardware design neuromorphic computing systems.

### Optically Stimulated Synaptic Devices

Inspired by optogenetics, optical signals have been incorporated into synaptic devices [90]. The use of optical signals offers a series of excellent characteristics, such as fast propagation speed, high bandwidth, and anti-crosstalk, all of which are expected to solve the challenges related to device integration and issues that electrically stimulated memristor-array-based synaptic devices could face. Moreover, optically stimulated synaptic devices use light signals as pulses to mimic synaptic functions and have provided a possibility for color identification in the artificial neural network [91].

In optically stimulated synaptic devices, the active materials selected mainly consist of oxide semiconductors [92–96], perovskite materials [97–99], nano-silicon [20, 100], graphene [101], two-dimensional sulfide [102], and so forth. Devices based on these materials have excellent photoelectric conversion performance [103–106]. Meanwhile, a decay for the conductance decreasing back to their initial values is expected in the devices so as to fulfill the requirement as synaptic devices. Therefore, the research on optically stimulated synaptic devices is essentially based on the exploration and development of optical memristors. Although most of them are volatile, the retention requirement can be relaxed for online learning, where the weights are updated frequently [107]. In this section, we mainly discuss the principles of related devices based on optical memristors for synapse simulation. These principles mainly include the ionization and dissociation of oxygen vacancies [92], capture and release of carriers by traps [94, 108, 109], and light-induced phase transition [17, 110].

#### Ionization and Deionization of Oxygen Vacancies

Oxide semiconductors have oxygen vacancies [111–113], which can be used to trap carriers. External light stimulation can lead to the ionization of oxygen vacancies; electrons then enter the conduction band and contribute to the conductance of materials. The recombination of electrons with ionized vacancies is difficult due to a relatively large potential barrier, thus indicating a good memory effect on the generated electrical signals. Therefore, oxide semiconductor-based devices were successfully used for mimicking synaptic functions [92, 114].

**Fig. 5:**
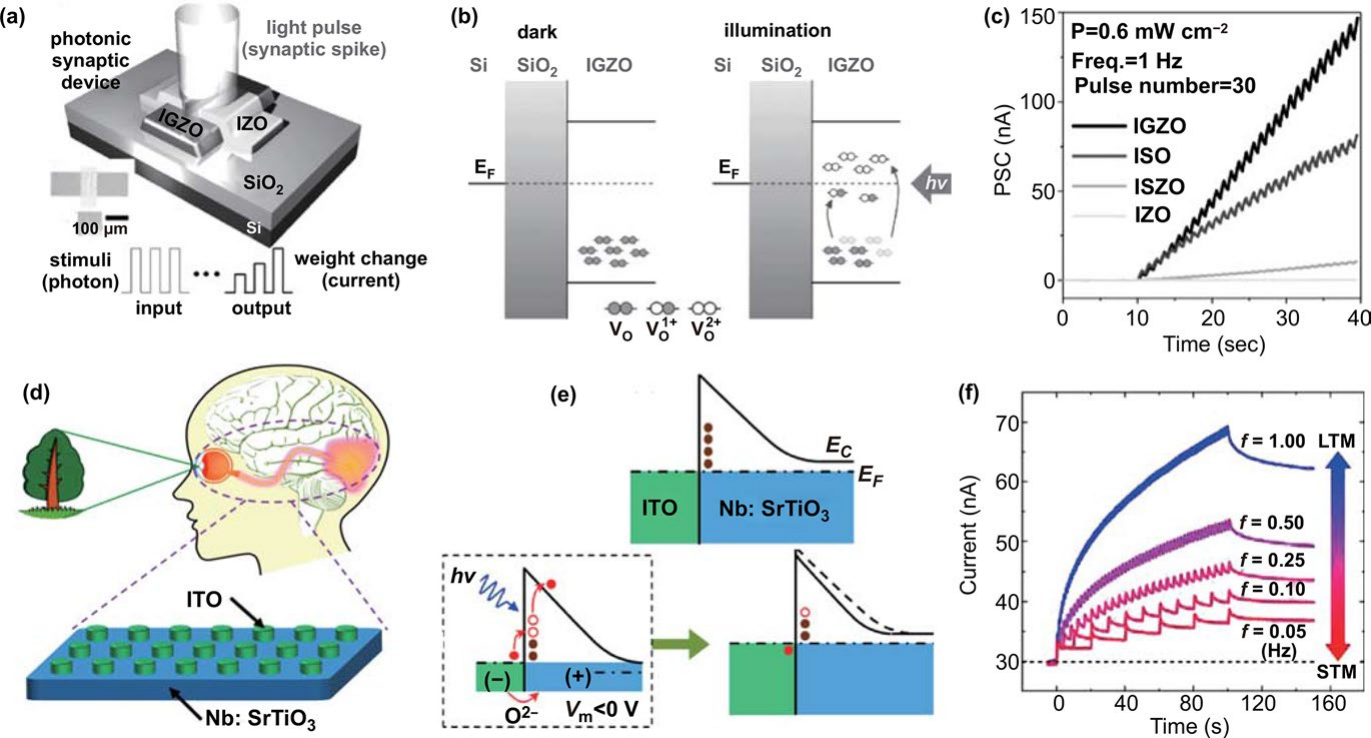
**a** Schematic of IGZO-based transistor synaptic device. **b** Schematic of the corresponding mechanism of the response stimulated by light pulse. **c** Postsynaptic current (PSC) trends of the IGZO, indium–strontium oxide (ISO), indium–strontium–zinc oxide (ISZO), and indium–zinc oxide (IZO)-based devices as the pulse numbers increased. **a**–**c** Reproduced with permission from Ref. [92]. **d** ITO/Nb:SrTiO_3_ Schottky heterojunction artificial optoelectronic synaptic devices. **e** Schottky barrier profile before and after light illumination accompanied by positive voltage stress. **f** A behavior of STM-to-LTM transition versus the increase of spike frequency. **d**–**f** Reproduced with permission from Ref. [25]

Figure 5a shows the synaptic device based on an indium–gallium–zinc oxide (IGZO) film studied by Lee et al. [92]. When the device received light stimulation, current signals were generated. This was related to the generation of photogenerated electrons. These electrons were mainly generated by the ionization of oxygen vacancies, in addition to the band (band edge)–band transition contribution in the device. As the number of optical pulses increased to 30, the postsynaptic current increased to 150 nA, thereby increasing the synaptic weight (Fig. 5b). To confirm that the expression of synaptic plasticity is related to the ionization and deionization of oxygen vacancies, Lee et al. [92] studied optically stimulated devices based on indium–strontium oxide (ISO), indium–strontium–zinc oxide (ISZO), and indium–zinc oxide (IZO). The working mechanisms of these devices and corresponding synaptic currents are shown in Fig. 5b, c. Photocurrents in these devices increased in the order of IZO → ISZO → ISO → IGZO (Fig. 5c). The observed photocurrents were found to be not reliable to the absorption coefficient and optical bandgap of these materials. We would like to mention that the change of decay time was consistent with the barrier heights for neutralization of ionized oxygen vacancies in these materials. Therefore, it was concluded that the conductive mechanism of the device is related to the process of oxygen vacancy ionization and deionization after light stimulation (Fig. 5b). To further understand the photocurrent variation of IGZO-based synaptic devices, Wu et al. [115] increased the concentration of oxygen vacancies by decreasing the concentration ratio of Ga to In in IGZO-based synaptic device and found this led to higher postsynaptic current (PSC) and enhanced persistent photoconductivity (PPC) effects. SnO_x_ could enable the extraction of weakly bound oxygen from IGZO [116]. Yu et al. [114] deposited a SnO_x_ film on IGZO-based synaptic device, which led to more oxygen vacancies in IGZO. Improved PSC and PPC effects were observed, indicating the role of oxygen vacancies during this process.

The ionization of oxygen vacancies also occurs at the interfaces or oxide surfaces [25, 117]. As can be seen in the work of Gao et al. [25], a simple indium tin oxide (ITO)/Nb:SrTiO_3_ Schottky heterojunction artificial optoelectronic synapse was fabricated (Fig. 5d). The traps at the interface of the heterojunction captured electrons, and the height and width of the Schottky potential barrier are large (Fig. 5e). In the case of light stimulation, the generated electrons left the interface, leaving behind some positively charged oxygen vacancy traps that can provide an additional potential to reduce the build-in electric field. At this point, the height and width of the Schottky potential barrier at the interface decreased, easing the transport of electrons and leading to an increased electrical performance. Mimicking of STM-to-LTM transition synaptic function is realized based on this process through the increase of spike frequency (Fig. 5f) or spike number in this device.

The change in synapse weight caused by optical pulses shows very good linearity [92]. This is related to the adsorption of a large number of electrons on oxygen vacancies and long decay time of the response stimulated by optical pulses. This linear-update LTP facilitates online learning efficiency in a neural network. These findings provide a basis for learning and information storage in neuromorphic computing. However, the device also presents shortcomings, such as high energy consumption and a failure to successfully realize the synaptic function of the IPSC.

#### Capture and Release of Carriers by Traps

In optoelectronic semiconductor devices, the electrons and holes are generated due to electrons (holes) jumping from the valence (conduction) band to the conduction (valence) band in response to optical pulses [118–122]. The generated carriers could be captured by traps in the devices [123]. These traps mainly include surface dangling bonds of nanoparticles [124], defects at the interface [125], potential wells, or barriers formed by a semiconductor bulk heterojunction [126]. These traps contribute to a slow decay of the photocurrents in the devices. This process has been used to realize the simulation of synaptic functions in recent years [95, 99, 108, 127, 128].

**Fig. 6:**
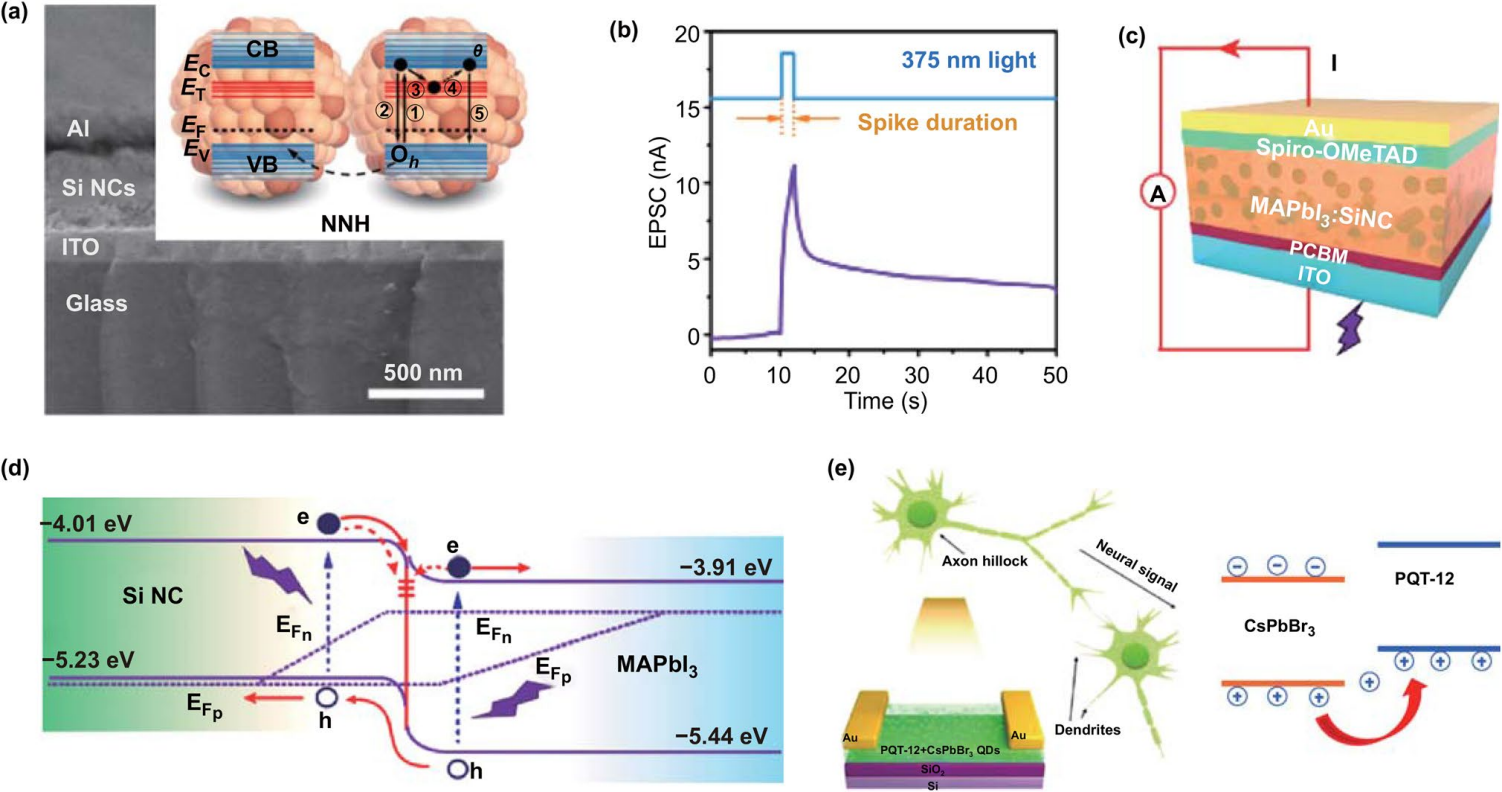
**a** Schematic of the silicon-based vertical two-terminal synaptic device; the inset shows the generation, capture, and release process of electrons. **b** EPSC of silicon-based vertical synaptic devices in response to 375-nm spike. **a**, **b** Reproduced with permission from Ref. [20]. **c** Schematic of an photoelectronic synaptic device with the multilayer structure of ITO/PCBM/MAPbI_3_:Si NC/Spiro-OMeTAD/Au on glass. **d** Schematic of band alignment between Si NCs and MAPbI_3_ and the behavior of the generation, trapping, and release mechanism of the carriers. Electrons and holes are denoted by filled and empty circles, respectively. Traps at the interface between MAPbI_3_ and Si NCs are indicated by short solid lines. **c**, **d** Reproduced with permission from Ref. [108]. **e** Schematic of the hybrid structure of CsPbBr_3_ and PQT-12-based synaptic device and its working mechanism. Reproduced with permission from Ref. [129]

Figure 6a shows a sandwiched synaptic device based on silicon nanocrystals (Si NCs) prepared by Tan et al. [20]. The boron-doped Si NC is a P-type semiconductor, and its surface has defects such as dangling bonds. When the device was stimulated by an optical pulse (375 nm), photogenerated carriers were produced, and the generated holes contributed to the conductance of the device (Fig. 6b). The photogenerated electrons were captured by dangling bonds at the surface in the film. The trapped electrons then escaped from the defects and recombined with holes, and the current gradually decreased. The process is shown in the inset of Fig. 6a. Synaptic functions, including PPF, STP-to-LTP transition, and SRDP, were mimicked. Defects at the interfaces of devices could also trap carriers. As can be seen in Fig. 6c, an optically stimulated synaptic device with a structure of ITO/PCBM)/MAPbI_3_: Si NC)/Spiro-OMeTAD/Au was prepared by Wen et al. [108]. A heterojunction between Si NC and MAPbI_3_ is formed in the optically active layer (Fig. 6d). The defects at the interfaces between Si NCs and MAPbI_3_ trap and release the photo-generated electrons and synaptic functions, such as EPSC, PPF, SNDP, and SRDP, were then successfully mimicked. The photocurrent was generated utilizing the photovoltaic effect, indicating the successful mimicking of synaptic functions without energy supply. Wang et al. [129] used potential wells to capture carriers and mimic synaptic functions in a horizontal synaptic device (Fig. 6e). A hybrid of inorganic perovskite CsPbBr_3_ and organic semiconductor poly(3,3-didodecylquarterthiophene) (PQT-12) was used as the active layer. The photo-generated holes in CsPbBr_3_ were trapped by the potential well, causing the current generated by the device to return slowly to its original state (right of Fig. 6e). Based on this condition, the device successfully mimicked the synaptic functions of EPSC, PPF, SNDP, and SRDP.

In these optically stimulated synaptic devices, semiconductor materials with excellent photoelectric conversion properties are used as the optically active layers, and various defects are introduced to trap and then release carriers, successfully realizing the simulation of neurosynaptic functions. Moreover, researchers successfully utilized the photovoltaic effect in some optically stimulated devices to achieve zero-power mimicking of synaptic functions. This endeavor can contribute to building low-energy neural networks. However, the difficulty of mimicking inhibitory synaptic functions remains a problem.

#### Optically Induced Phase Transition

The difficulty of mimicking IPSC in optically stimulated synaptic devices is due to the generation of carriers in response to optical pulses in the materials. This makes implementing erasing programming almost impossible with optical pulses. Phase transition can be regulated by a light power supply [70, 130]. Phase transitions in these materials include transformations between semiconductor and metallic states [17], and between crystalline and amorphous states [131]. The conductivity and the light absorption properties of the materials during transformations would then be changed significantly. Meanwhile, the states after phase transition are expected to have excellent memory effects. Researchers have utilized these characteristics for neuromorphic computing as discussed below.

**Fig. 7:**
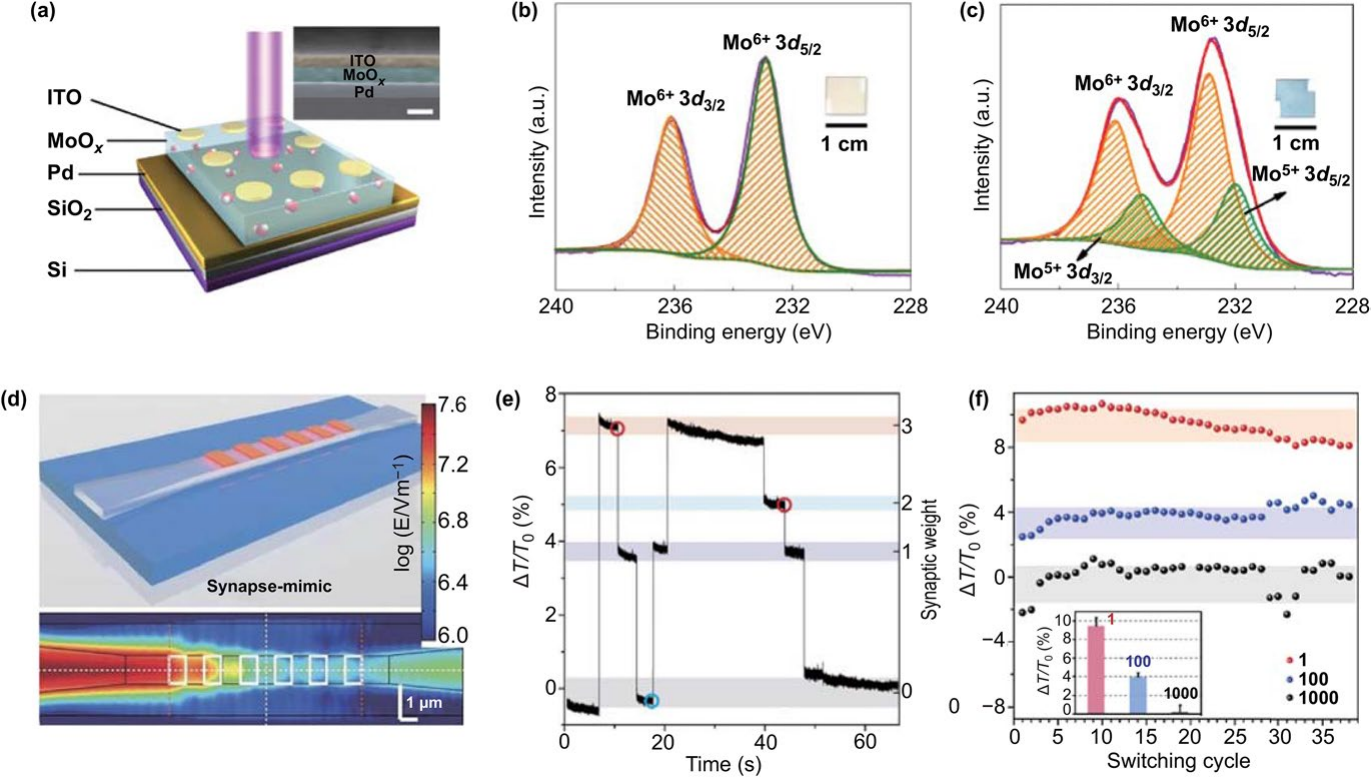
**a** Schematic of MoO_x_-based device. **b** Narrow scans of the Mo 3d peak for the MoOx layer before UV illumination. **c** Narrow scans of the Mo 3d peak for the MoOx layer after UV illumination. **a**–**c** Reproduced with permission from Ref. [17]. **d** Top: Photonic synapse with synapse-mimic design: a tapered waveguide with six discrete Ge_2_Sb_2_Te_5_ (GST) islands (1 mm × 0.8 mm each). **e** Change of synaptic weight in the device during switching with the relative transmission change (ΔT/T_0_) recorded. **f** Cycle-to-cycle weight changes. The inset: Statistical analysis of different weights at different pulse numbers. **d**–**f** Reproduced with permission from Ref. [110]

One principle of synaptic devices based on optically induced phase transition is related to the current response stimulated by optical pulses. As shown in Fig. 7a, Zhou et al. [17] reported this mechanism in a vertical two-terminal structure comprising ITO/MoO_x_/Pd. When the external UV light stimulation was incident from the ITO end, electron–hole pairs were generated in MoO_x_. The photogenerated holes reacted with absorbed water molecules, and electrons were transferred into the MoO_x_. This process led to a phase transition from semiconductor to metallic state, in the process of which the Mo oxidation state changed from Mo^6+^ to Mo^5+^. Figure 7b, c shows the corresponding X-ray photoelectron characterization results of Mo 3d core spectra of MoO_x_ before and after the UV illumination and proved the assumption for this mechanism. When continuous light stimulation was applied, the current gradually increased. The photocurrent increased from 90 to 170 mA cm^−2^ as the light intensity was increased from 0.22 to 0.88 mW cm^−2^. More interestingly, the photocurrent, in response to different light intensities, decreased slightly in 300 s and exhibits good memory. These properties ensure the realization of learning and memory function with optically stimulated synaptic devices.

Optical pulses can induce a phase transition of Ge_2_Sb_2_Te_5_ (GST) between amorphous and crystalline states [131], resulting in light absorption properties changes after the phase transition. Cheng et al. [110] built a purely photonic synapse based on this principle. The structure of this synapse is a waveguide (Si_3_N_4_) with discrete GST on top (Fig. 7d). The synaptic weight was expressed as relative light transmission change ΔT/T_0_ through the waveguide (Fig. 7e), $\Delta T = T - T_{0}$, where *T*_0_ is the transmission in the crystalline state of PCM, and *T* is the light transmission after pulses. In the crystalline state, the PCM is more absorptive, leading to strong attenuation of the passing optical signal in the waveguide. On the other hand, the absorption is obviously reduced in the amorphous phase of PCM. This leads to that the transmission through the waveguide is not reduced significantly. Since the phase transition of the PCM was induced by the high-energy optical pulses, a highly effective way of regulating light transmission was realized through controlling the number of applied excitation pulses. Therefore, the inhibitory synaptic function could be mimicked in the photonic synapse (Fig. 7e). This photonic synapse exhibited a small cycle-to-cycle variation of 0.77% in its weight changes even after 38 pulses (Fig. 7f). GST is considered to be an ideal candidate as a purely photonic synapse used in neuromorphic computing, due to prominent scalability, multiple programmable resistances and low device-to-device variation. Stimulated by light, the number of carriers in the optically stimulated synaptic devices increases in response to external optical pulses. This can cause a weight change of synaptic devices and realize the mimicking of the EPSC (LTP). Given that carriers are always generated when such synaptic devices are exposed to optical pulses, the mimicking of IPSC (LTD) function in the optically stimulated synaptic devices is difficult. A unidirectional method can solve the challenges of the ANNs in which the devices only have the function of LTP [132].

However, the realization of a synaptic device that possesses both LTP and LTD functions is significant for improving the learning efficiency and simplifying the ANNs. Theoretically, an infrared thermal effect can be considered to deteriorate the electrical performance of the devices and mimic the depression properties. However, many drawbacks based on such a thermal effect may exist, and thus, improved means of regulating the electrical performance are needed. In recent years, researchers have used the synergistic effects of electrical and optical pulses to regulate the electrical performance of their devices and realized the mimicking of synaptic functions of LTP and LTD in a single device [133].

### Photoelectric Synergetic Synaptic Devices

As discussed above, electrical pulses and optical pulses both can regulate the properties of the materials, leading to construction of electrically stimulated and optically stimulated synaptic devices, respectively. Optical pulses can not only produce electron–hole pairs [134] but also regulate the formation of vacancies or ions in some materials [90], while electrical pulses can induce the movements of carriers and ions [32, 33, 135]. Optical pulses and electrical pulses both can induce the phase transitions of the materials [70, 74]. Besides, electrical pulses can induce the polarization of the ferroelectric materials, while optical pulses may induce the photocurrent response due to a photovoltaic effect of ferroelectric materials [64, 136]. These conditions promote the development of photoelectric synergetic synaptic devices, which utilize optical and electrical pulses to control the electrical performance of devices through their synergistic effects [27, 94, 133]. They contain electrically assisted optical stimulation and optically assisted electrical stimulation. For electrically assisted optically stimulated devices, photogenerated electrons may be trapped by various trap centers as discussed above. Carriers can be erased by applying a bias voltage to realize the mimicking of IPSC (LTD). At the same time, in optically assisted electrically stimulated devices with a horizontal structure, optical pulses can be used to control the formation and annihilation of ion vacancies that can promote the mimicking of IPSC (LTD); in optically assisted electrically stimulated devices with a vertical structure, light may reduce the energy required for ion migration, which plays an important role in reducing electrical-stimulation energy consumption and increasing the range of weight changes. Here, we mainly discuss synaptic devices based on these two aspects.

#### Electrically Assisted Optical Stimulation

**Fig. 8:**
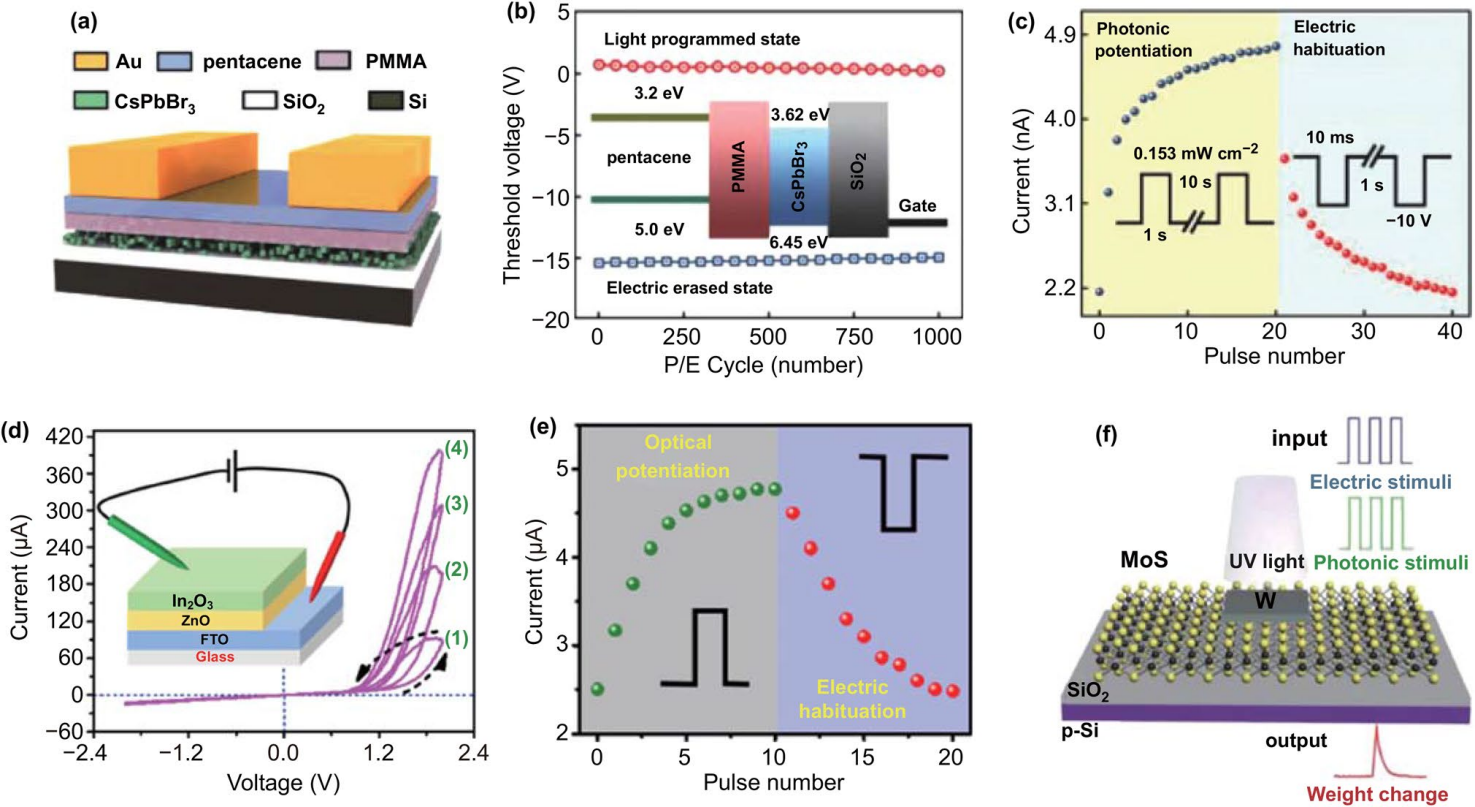
**a** Schematic of the CsPbBr_3_ quantum dot-based synaptic device. **b** Schematic of the device energy band diagram. **c** Photonic potentiation in response to optical pulses and depression in response to electrical pulses under dark condition. **a**–**c** Reproduced with permission from Ref. [137]. **d** In_2_O_3_/ZnO/FTO-based synaptic device and its *I–V* characteristic at different cycles. **e** Current changes for the synaptic devices in response to excitatory optical pulses and inhibitory electrical pulses. Top and bottom insets show the shape of the pulses. **d**, **e** Reproduced with permission from Ref. [94]. **f** Schematic of an optoelectronic synergetic synapse device based on monolayer MoS_2_. Reproduced with permission from Ref. [27]

For electrically assisted optically stimulated devices, electrical stimulation is mainly used to erase the trapped carriers (ions) caused by optical stimulation and mimic the synaptic function of IPSC (LTD). Han et al. [137] prepared a transistor-type synaptic device based on a hybrid structure of inorganic perovskite and pentacene (Fig. 8a). In this structure, pentacene was used as the channel material and perovskite as the photosensitive material. It exhibited a type-II heterojunction (Fig. 8b). When the device was stimulated by light, the carriers were generated in the inorganic perovskite. The holes entered the pentoxide and contributed to the conductance of the device. The electrons were trapped in the perovskite. Thereby, the mimicking of LTP synaptic function was successfully realized (Fig. 8c). The energy band structure was adjusted by applying a bias on the gate, allowing electrons to be slowly released and recombine with holes in the pentoxide to achieve mimicking of IPSC function (Fig. 8c). Figure 8d shows a device with a vertical two-terminal In_2_O_3_/ZnO/FTO structure [94]. The pink I-V curve shows a hysteresis effect related to the trapping of electrons at the interface of In_2_O_3_ and ZnO. They stimulated the generation of carriers by UV light and utilized defects at the interface to trap electrons and mimic the synaptic function of EPSC. The electrons were erased by reverse electrical stimulation to realize the simulation of inhibitory synaptic function (Fig. 8e). Similarly, He et al. [27] constructed a two-terminal synaptic device with a structure of W/MoS_2_/p-Si (Fig. 8f). MoS_2_ and p-Si formed a p-n heterojunction in the device. The conductivity of MoS_2_, in response to UV light, was enhanced by the generation of carriers. The excitatory postsynaptic function was then successfully mimicked. When a positive electrical stimulus was applied to the structure (p-Si is connected to the positive bias), a portion of the carriers were captured by defects (Si–O dangling bonds) at the interface. This worsened the conductivity realizing the mimicking of IPSC function. Besides, 2D material-based transistor synaptic devices (such as a MoS_2_/PTCDA hybrid heterojunction synapse [138] and an h-BN-encapsulated MoS_2_ synaptic transistor [139]) were constructed. Carriers are generated in PTCDA or h-BN in response to optical spikes and then transferred to the MoS_2_ channel in these devices. Various synaptic functions are successfully mimicked through combing optical spikes and electrical modulation. It is worth mentioning that plenty of work has been done based on electrical spikes to regulate the distribution of carriers in the devices and contribute to the mimicking of IPSC [101, 114, 115, 140, 141]. In these structures, photoelectric synergistic effects are utilized to control the electrical properties. Advantages of both electrical and light pulses could be utilized during the operation and realize the mimicking of LTP and LTD synaptic functions in one device, thereby promoting the development of neuromorphic computing.

#### Optically Assisted Electrical Stimulation

**Fig. 9:**
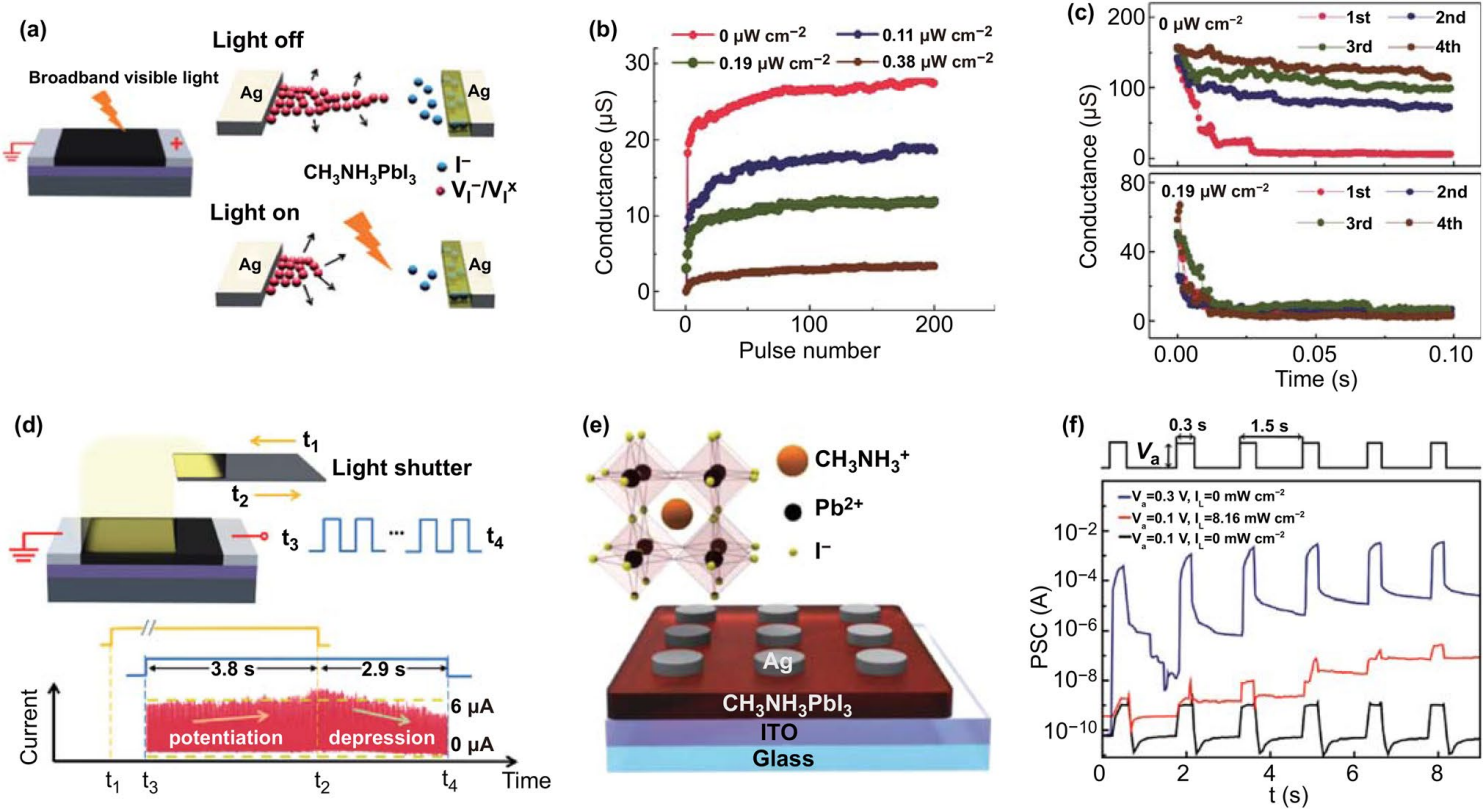
**a** Schematic of the device and the influence of light on ion diffusion. **b** Changes in conductance in response to optical spikes versus the pulse number at different power densities. **c** Retention curves as the device is repeatedly stimulated by electrical spikes in the conditions of dark and light illumination. **d** Mimicking of LTP and LTD synaptic functions in the conditions of dark and light illumination. **a**–**d** Reproduced with permission from Ref. [90]. **e** Schematics of the Ag/MAPbI_3_ (OHP)/ITO synaptic device and OHP structure. **f** Postsynaptic currents at different pulse conditions. **e**, **f** Reproduced with permission from Ref. [133]

For an optically assisted electrically stimulated synaptic device, light is used as a supplementary way to regulate the device. For example, Zhu et al. [90] studied synaptic devices with a horizontal structure based on organic–inorganic hybrid perovskite (MAPbI_3_) (Fig. 9a). Under external electrical stimulation, iodine vacancies were formed while optical pulses were introduced, evidently, preventing the formation of iodine vacancies. Therefore, light influenced the change of the resistance state of MAPbI_3_. As the number of electrical pulses increased in the dark condition, the electrical performance of the device gradually improved (Fig. 9b). When the light intensity was gradually increased from 0.11 to 0.38 μW cm^−2^, the conductivity gradually deteriorated from ~ 20 to ~ 5 μS. This phenomenon was attributed to light irradiation which inhibited migration of ions and accelerated the recombination of ions and vacancies.

Light also affects the decay time of electrical performance. As shown in Fig. 9c, in the absence of light stimulation, the decay time increased with an increase in the number of electrical stimulations. However, once light was irradiated (0.19 μW cm^−2^), the decay time significantly reduced and became independent of the changes in the number of scans. These changes revealed that electrical stimulation can induce ion migration and enhanced conductivity and that light can prevent ion migration or the formation of vacancies, accelerated the recombination of ions and vacancies, and reduced conductivity. These findings ensured that the mimicking of important synaptic functions, such as LTP (dark condition) and LTD (1.29 μW cm^−2^), can be realized through combined electrical and optical stimulations in one device (Fig. 9d).

Figure 9e shows a vertical Ag/ MAPbI_3_/ITO synaptic device constructed by Ham et al. [133]. Through external electrical stimulation, the movement of iodine vacancies was regulated to form conductive filaments in the device, and mimicking of synaptic functions was realized. In addition, optical stimulation induced ion migration. This phenomenon may be related to the changes in the built-in electric field of the device and the activation energy of ion migration due to the photogenerated carriers, which is contrary to the performance caused by optically assisted devices based on MAPbI_3_-based horizontal device as discussed above. The source of this difference and its mechanism are still unclear, and further research is needed. Light promoted ion migration in the vertical two-terminal device and ensured ion migration with a small voltage in the device (Fig. 9f). In addition, significantly more ions can migrate in the device compared with the case under dark conditions (Fig. 9f), leading to a larger postsynaptic current under the condition of light exposure. These ensure the mimicking of linear LTP and LTD synaptic functions with a small energy consumption in the device.

## Application of Synaptic Devices

Synaptic devices have mimicked biological synaptic functions, such as STP/STD, LTP/LTD, SRDP, and STDP. These functions have shown many application areas, which mainly include pattern recognition, logical operations, associate learning, filtering, and so on.

### Pattern Recognition

**Fig. 10:**
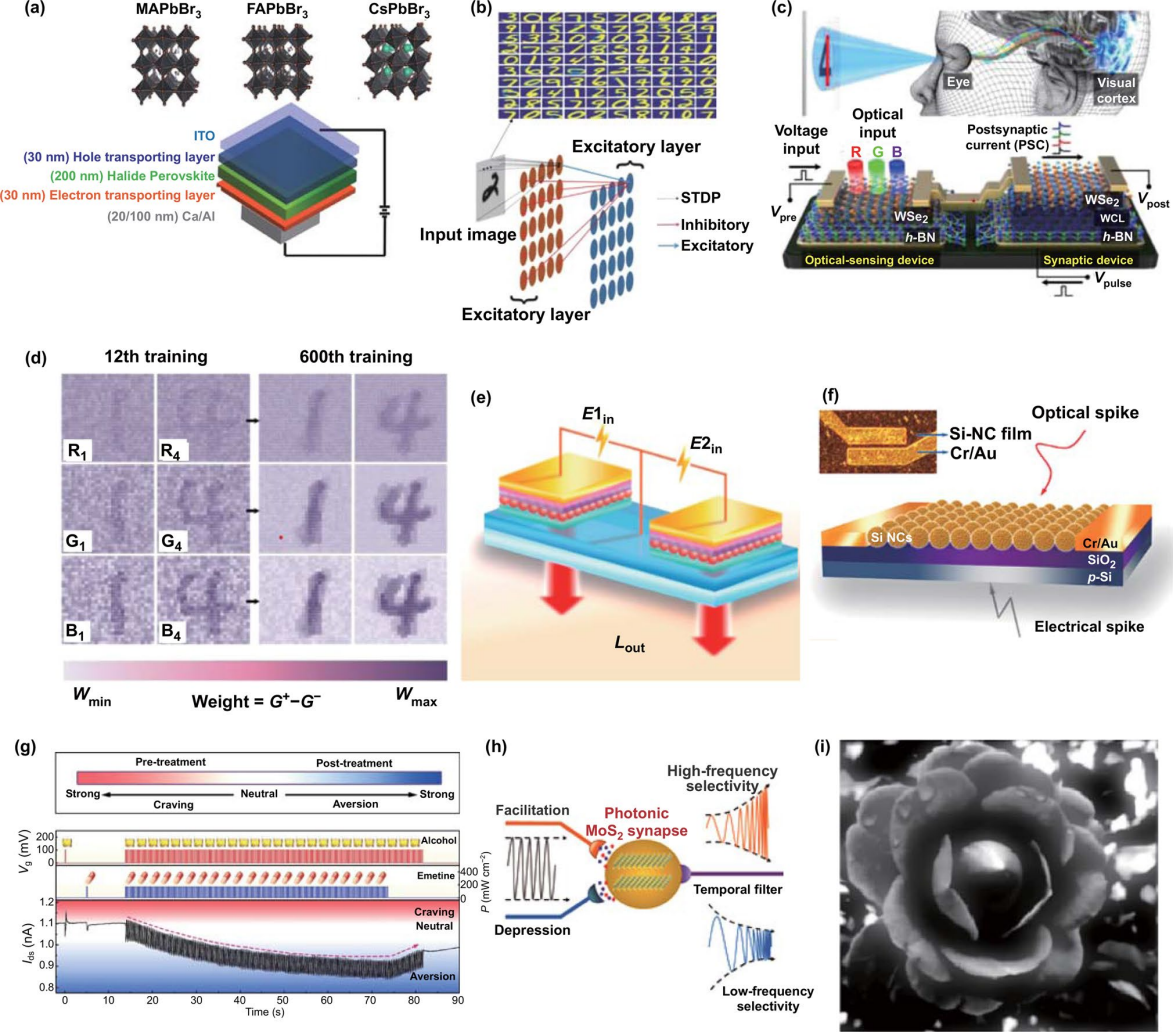
**a** Synaptic devices based on halide perovskites, including MAPbBr_3_, FAPbBr_3_, and CsPbBr_3_. **b** Handwritten numeral recognition through the neural network based on synaptic devices. **a**, **b** Reproduced with permission from Ref. [143]. **c** A optic-neural synaptic device based on the h-BN/WSe_2_ heterostructure. **d** Synaptic values change versus the increase of the training number. **c**, **d** Reproduced with permission from Ref. [91]. **e** Electroluminescent synaptic devices based on Si NCs. Reproduced with permission from Ref. [30]. **f** Si NC-based transistors synaptic device. **g** Aversion learning of the devices used for the treatment of people addicted to alcohol. **f**, **g** Reproduced with permission from Ref. [100]. **h** Photonic MoS_2_ synapse as a temporal filter. Reproduced with permission from Ref. [102]. **i** Image of a flower after sharpening with a high-pass filter. Reproduced with permission from Ref. [108]

STDP synaptic function is considered the most important learning mechanism of the brain and plays a particularly important role in pattern recognition [45, 133, 142, 143]. For example, John et al. [143] constructed an electrically stimulated synaptic device based on perovskites (MAPbBr_3_, FAPbBr_3_, or CsPbBr_3_) and successfully realized the mimicking of STDP synaptic function (Fig. 10a). On the basis of this synaptic function, a two-layer neural network was constructed to recognize handwritten digits from the Modified National Institute of Standards and Technology (MNIST) dataset (Fig. 10b). Through image training, the neural network achieved 80.8% accuracy of recognition. However, the image recognition implemented by John et al. was just realized with general software algorithms [144]. A perceptron classifier implemented with a realistic 2 × 10 titanium dioxide passive memristive crossbar circuit was experimentally demonstrated in 2013 [145]. This work presented a proof-of-concept demonstration for hardware memristor-based ANNs. Later, a 1T1R-array structure-based hardware neural network achieved a relatively high recognition rate for grayscale face classification [146]. However, further investigation is still needed for an extensively practical use of hardware-based networks. Seo et al. [91] fabricated a photoelectric synergetic synaptic device through integrating a synaptic device with an optical-sensing device on the same h-BN/WSe_2_ heterostructure (Fig. 10c). The device responded differently to the wavelengths of red (R), green (G), and blue (B) light. The weight control layer (WCL) formed by O_2_ treatment on h-BN was used for trapping and de-trapping of electrons for mimicking plasticity. Figure 10d shows the synaptic weight values in the optical neural network after the 12^th^ and 600^th^ training epochs, indicating an influence of epoch number on recognition effect. This optical neural network successfully implemented the recognition task for the colored and color-mixed numbers (1 and 4) with a recognition rate of 90%, indicating significant potential for application in color-mixed number recognition based on photoelectric synergetic synaptic devices. Image or number recognition is thought to be one of the most important applications for synaptic devices. Currently artificial synaptic devices can reach a high accuracy rate in digital recognition and promote the application of brain-like computing.

### Logic Operation

Introducing logic operation function into synaptic devices enhances their ability to process information [11, 147]. As shown in Fig. 10e, Pi et al. [30] constructed a silicon-based crystal electroluminescent neurosynaptic device; the logical operations of “AND” and “NOT” were performed based on the devices. The principle is based on two devices that are excited with two electrical stimuli with voltages of E1_in_ and E2_in_. The devices emit light in response to external electrical spikes, and the total luminous power is considered the output of the “AND” and “NOT” gates. The threshold was set in advance. During the operations, the luminous power value was measured after the stimulation of electrical pulses, thereby realizing the logical operations of “AND” and “NOT”. The logical operations of “NAND” and “NOR” were also realized in the devices by measuring the changes in their resistance states. Meanwhile, Hao et al. [148] fabricated optically stimulated synaptic device based on a hybrid of DPPDTT and CsPbBr_3_. Logic operations of “AND” and “OR” were realized by measuring the photocurrents with optical stimuli using different light wavelengths and intensities. These independent logic gate devices are expected to be integrated into logic circuits and will play a significant role in complex logic operations.

### Associate Learning

Associate learning describes the repetitive occurrence of two inputs in short time ranges and formation of brain connections, such as conditioned reflex, and other behaviors [149, 150]. The Pavlov’s Dogs experiment is a good example of conditional reflex and the behavior that can be realized in neuromorphic computing [151]. Two excitatory stimulation modes are required to simulate this behavior with synaptic devices. For aversion learning, synaptic devices need to have both excitatory and inhibitory modes [100]. Figure 10f shows the transistor-type synaptic device based on Si NCs [100]. When continuous electrical stimulation is applied through the gate, the device produces a long-term potentiated postsynaptic current, and when optical stimulation is applied, the device produces a long-term depressed postsynaptic current. Therefore, aversion learning can be simulated based on this property. Aversion learning is usually used in the treatment of people with alcohol addiction (Fig. 10g). During a person’s initial addiction to alcohol, the brain transitions into an excited state after drinking. To treat this behavior, patients need to drink alcohol first and then vomit by taking emetine. After repeated drinking and taking emetine, the patients become more averse to alcohol. The application of electrical stimulation to the synaptic device followed by that of light stimulation is similar to the process of the patient drinking alcohol and taking emetine, which can realize the simulation of aversion learning (Fig. 10g).

### Filtering

Filtering is an important function of brain synapses [9]. This process is related to the synaptic function of SRDP, which is important for the construction of an artificial neural network which realizes filtering function. This function has been realized in both optically stimulated and electrically stimulated synaptic devices [102, 108, 152]. Figure 10h shows the temporal filter function of a synaptic device constructed by Jiang et al. [102] based on a MoS_2_ horizontal structure. For a given 50-Hz continuous light stimulation, the EPSC of the synaptic device increased, whereas the IPSC decreased when the frequency was 2 Hz. On this basis, synaptic devices can be used as high-pass and low-pass filters. Figure 10i shows a specific application of a synaptic device as a high-pass filter in sharpening an image at the cutoff frequency of 4.8 Hz, making the image clearer [108]. These indicate synaptic devices can potentially realize the effective transmission of information through their corresponding filtering characteristics in an artificial neural network.

## Perspectives

Plenty of functional materials have been used to fabricate synaptic devices in recent years. These synaptic devices have various performance indicators, such as pulse width, device size, energy consumption, completeness of synaptic functions achieved, dynamic range, the number of states, linearity of weight change, retention, and endurance [28]. Each of these parameters should meet their corresponding requirements as synapses in neural networks for efficient neuromorphic computing. A list of representative synaptic devices, including part parameters, are shown in Table 1.

**Table 1:** Performance metrics of representative synaptic devices (2 T:2 terminals, 3 T:3 terminals)

Stimulation	Materials (structure)	Mechanism	Size (diameter or width × length)	Energy consumption	Time	LTP (dynamic range/LTD (dynamic range/STDP
Electrical	Ag/GeS_2_/W (2 T) [44]	Ion migration	200 nm	1800–3100 pJ	500 ns	√(1000)/√(1000)/√
	Ag/a:Si/W(2 T) [60]	Ion migration	100 nm	720 pJ	300 μs	√(15)/√(7)/√
	2D h­BN (2 T) [85]	Ion migration	10 µm × 10 µm	–	20 μs	√(20)/√(20)/√
	PCMO (2 T) [154]	Ion migration	150 nm	6–600 pJ	10 μs	√(1000)/√(1000)/√
	IGZO/SiO_2_ (3 T) [155]	Ion migration	150 µm × 1 mm	0.23 pJ	150 ms	√(4)/–/–
	GST (2 T) [74]	Phase change	300 nm	121–1552 pJ	50 ns	√(10)/√(400)/√
	2D Li^+^/MoS_2_ (3 T) [87]	Phase change	7 µm × 21 µm	–	1 ms	√(5.6)/√(5.6)/–
	BFO/CCMO (2 T) [39]	Ferroelectric	180 nm	–	100 ns	√(20)/–/√
	Pb(Zr,Ti)O_3_ (3 T) [38]	Ferroelectric	5 μm × 200 µm	–	1 ms	√(4.7)/√(5)/√
	PVPy/Au NPs(2 T) [42]	Trap	5 µm × 5 µm	–	100 ms	√(7.5)/√(7.5)/–
	AuNPs/pentacene(3 T) [79]	Trap	5 µm × 1000 µm	5 µJ	2 s	√(16)/√(16)/–
	2D WSe_2_ and MoS_2_ (3 T) [88]	Trap	5 μm × 20 µm	–	20 ms	√(23)/√(23)/–
Optical	Nb:SrTiO_3_ (2 T) [25]	Ionization of Oxygen vacancy	100 μm	–	0.5 s	√(2.3)/–/–
	IGZO (3 T) [92]	Ionization of oxygen vacancy	180 μm × 70 μm	–	5 s	√(3.7)/–/Symmetry
	MoO_x_ (2 T) [17]	Phase change	9 μm × 9 µm	4.8 pJ	2 s	√(2)/–/–
	Si NCs (2 T) [20]	Trap	2 mm × 2 mm	0.14 nJ	2 s	√(6.3)/√(3.3)/–
	MAPbI_3_/Si NCs (2 T) [108]	Trap	30 μm × 30 μm	0 fJ	50 ms	√(3.4)/–/–
	2D (PEA)_2_SnI_4_ (3 T) [127]	Trap	1 mm × 1 mm	–	1 ms	√(9.8)/–/–
Synergism	Ag/MAPbI_3_/ITO (2 T) [133]	Ion migration	100 µm	1.9 nJ	0.4 s	√(26)/√(26)/–
	ZnO/In_2_O_3_ (2 T) [94]	Trap	–	0.2 nJ	1 s	√(1.9)/√(1.8)/–
	MAPbI_3_/Si NCs (3 T) [97]	Trap	25 µm × 500 µm	1 pJ	200 ms	√(2.5)/√(4.6)/–
	CsPbBr_3_/pentacene (3 T) [137]	Trap	0.50 mm × 1 mm	1.4 nJ	1 s	√(2.2)/√(1.7)/–
	2D MoS_2_ (3 T) [139]	Trap	10 µm × 15 µm	–	100 ms	√(100)/√(100)/–

In the synaptic device context, the dynamic range of the synaptic devices refers to the ratio of the current value of the high conduction state to that of low conduction state. The dynamic range of the synaptic weight for the synaptic devices should be at least 100, which accords with the requirement of biological synapses [14]. At present, the dynamic range in response to electrical and optical pulses has received some progress and can achieve the requirement for some devices. However, further works are needed to fulfill the broad applications for neuromorphic computing. For externally continuous pulses, numerous states (20–200) exist between the lowest and highest synaptic weights for the synaptic devices, which reflect the nature of biological cumulative effects on external pulses [28]. In addition, the change in synaptic weights is expected to be linear with the number of pulses, which is important for the information processing in artificial neural network [14, 153]. Currently, the number of states and linearity of the synaptic weights are inferior for most electrically and optically stimulated synaptic devices. Considerable efforts should be devoted to improving these properties by, for example, adding ion-migration-blocking layers and increasing the abilities of trapping carriers in the devices.

Regarding the retention and endurance performances for the traditional crossbar-array memristors, the retention time can reach up to the year level, and the endurance can be in the range of 10^8^–10^13^, which can meet the application requirements in neuromorphic computing [28]. Research into neurosynaptic devices based on optical pulses started relatively late, and retention and endurance performances are inferior so far but is expected to make progress in the near future. This calls for exploration and applications of various optical memristors in neuromorphic computing. We should mention that requirements for the retention time of synaptic devices mainly depend on their applications [15]. If neural networks are trained online, the weights in such networks are updated rapidly and the retention performance of the synaptic devices is less strict [14].

The size of a synaptic device is one of the key parameters which can determine the degree of integration. The smaller the device size, the higher the ease of integration. The density of synapses in the human brain neural network is about 10^9^ mm^−3^, and the physical size of each synapse is about 20 nm [28]. The size of the synaptic device should also be within this magnitude range to build an artificial neural network. At present, the physical size of resistive-change and phase-change materials can be reduced to below 10 nm [15], which is conducive to the construction of artificial synaptic devices with the same magnitude as biological synapses. As shown in Table 1, the size of electrically stimulated synaptic devices can reach below 100 nm. Optically stimulated and photoelectric synergetic synaptic devices are still relatively large [20], with sizes remaining above the micron level (Table 1). This large size is related to the weak sensitivity and response of the devices to external optical pulses.

Energy consumption is an important parameter for mimicking synaptic functions as well. The human brain neural network consists of ~ 10^13^ neurons and ~ 10^15^ synapses. Under normal circumstances, 1% of neurons and synapses in the brain’s neural network are activated. Therefore, the entire power consumption of the brain is about 20 W, and the power consumption of each synapse in the brain is about 10^–13^ W. If the time of each synaptic event is about 100 ms, the energy loss is 10 fJ. Table 1 shows energy consumption of various representative synaptic devices thus far. Most existing electrically stimulated or optically stimulated synaptic devices have energy consumption above a picojoule (pJ) and cannot meet the high-performance computing requirements for energy consumption below 10 fJ.

Synaptic devices also should meet requirements for external pulse width. In general, the pulse width is expected to be *τ* < 1 ms [28]. The shorter the pulse width is, the higher the learning efficiency may be. At present, the pulse width of electrically stimulated devices is on the order of microseconds. However, the pulse width of optically stimulated synaptic devices is relatively long (Table 1) and cannot meet the needs of high-efficient computing.

The size and programming time of electrically stimulated synaptic devices are superior to that of optically stimulated devices, while energy consumption is still large for both electrically stimulated and optically stimulated devices. Electrically stimulated synaptic devices can mimic relatively complete synaptic functions, including EPSC, IPSC, LTP, LTD, and STDP, whereas for optically stimulated synaptic devices, the mimicking of depression-related synaptic functions has always been difficult to realize (Table 1). Such difficulty is due to enhanced conductivity of the optically stimulated devices arising from generation of carriers in response to external optical pulses, resulting in the difficulty to mimic synaptic functions, such as IPSC. Synaptic devices should have an IPSC synaptic function, which is an inevitable requirement for realizing the mimicking of important synaptic functions, such as LTD and asymmetric STDP. Thus, the coordinated regulation of optical and electrical stimulation to change the electrical properties of devices and achieve synaptic functions, such as IPSC/LTD, should become a research trend.

Considering the actual performances, great efforts should be put into improving photoelectric-conversion and memristive properties for the studies of nanoscale synaptic devices. Besides, synaptic devices based on phase transition, carriers trapping, ions migration and ferroelectric effects are worth attracting more attention since these are very possible for mimicking LTD and STDP synaptic functions based on photoelectric synergetic effect. In addition, exploring new proof-of-concept architectures as well as principles based on optical and electrical signals is expected in the devices for future-efficient neuromorphic computing. These could address drawbacks such as poor LTP linearity as well as high energy consumption for electrically and optically stimulated synaptic devices. These could also solve the challenges of device size and programming time as well as mimicking IPSC functions for optically stimulated synaptic devices. Furthermore, it could be imperative to develop new algorithms or neural networks that utilize the unique properties of these memristive devices for neuromorphic computing.

A construction of hardware neural networks for the neuromorphic computing systems is essentially the integration of memristive arrays. At present, given the parasitic current of crossbar-array-based memristive systems [156], the anti-interference capability could be extremely poor in hardware-based neural networks. This is a significant challenge in the applications of the system, leading to possible read errors and increased energy consumption during the training process. To address the parasitic current issue, a select device such as a transistor or a selector connected in series with a memristor (1T1R or 1S1R arrays) is required. However, it should be noted that all the passive crossbar, 1T1R and 1S1R arrays face some common challenges such as the IR drop caused by long electrode wires in large-scale arrays, which indicated the requirement of further considerations. Besides, construction of synaptic devices with excellent rectifying properties could be considered and is critical for the development of future neuromorphic computing.

## Conclusions

This study analyzed and discussed synaptic devices based on pulses of optical and electrical signals. In accordance with the signal stimulation mode, the devices were divided into electrically stimulated, optically stimulated, and photoelectric synergetic synaptic devices. This paper discussed in detail the respective working principles of various synaptic devices, progress, and applications. The essential mechanism of the synaptic devices is based on the properties of memristive systems. Except for ferroelectric and metal-ion-migration-based synaptic devices, which are only related to electrical pulses, all other mechanisms such as phase transition, capture and release of carriers, as well as oxygen and halide ions migration are related to both electrical and optical pulses. It is worth mentioning that the photovoltaic effect exists in ferroelectric materials. Optical pulses are expected to stimulate the ferroelectric materials for mimicking synaptic functions.

Electrically stimulated synaptic devices have many advantages, such as complete synaptic function simulation, scalability of the devices, and good endurance property. However, the largest drawbacks of electrically stimulated synaptic devices are high energy consumption, limited number of weight changes, and poor linearity of the LTP behavior. Further device engineering is obviously needed to address these issues. In optically stimulated synaptic devices, optical signals have the advantages of high bandwidth and fast propagation speed and can directly simulate vision for color recognition. However, the device area and width of optical pulses are still large, and energy consumption is high. Furthermore, the mimicking of IPSC function is difficult to realize in these devices. Further investigations into improving these synaptic properties and increasing the linearity of LTP behaviors by seeking excellent photoelectric conversion materials and frameworks are needed for the applications of these synaptic devices.

Recently, photoelectric synergetic synaptic devices have demonstrated some progress in mimicking IPSC/LTD functions and decreasing energy consumption. This provides a possibility that a device could possess both LTP and LTD functions, which utilize the advantages of both electrical and optical signals to regulate the properties of devices. Therefore, emerging materials and architectures based on excellent optical and electrical properties (e.g., perovskite and 2D-layered materials) deserve investigation for photoelectric synergetic synaptic devices. Due to the needs of the integration, synaptic devices with rectify properties are worth studying by exploring various heterojunction devices. These are expected to significantly contribute to the large-scale deployment of neuromorphic computing.
